# Additive effects of obesity and loneliness on C-reactive protein

**DOI:** 10.1371/journal.pone.0206092

**Published:** 2018-11-15

**Authors:** Gregory Pavela, Young-il Kim, Sarah-Jeanne Salvy

**Affiliations:** 1 Department of Health Behavior, University of Alabama at Birmingham, Birmingham, Alabama, United States of America; 2 Division of Preventive Medicine, University of Alabama at Birmingham, Birmingham, Alabama, United States of America; 3 Department of Medicine, Cedars Sinai Medical Center, Los Angeles, California, United States of America; University College London, UNITED KINGDOM

## Abstract

Obesity and loneliness are associated with C-reactive protein (CRP), a predictor of cardiovascular disease. It is unknown whether the co-presence of obesity and loneliness is associated with additional risk for clinically elevated CRP. The present study thus examines their independent and combined effects on elevated CRP in older adults. Data come from 10,912 respondents who completed the 2008 and 2010 waves of the Health and Retirement Study. Loneliness was measured using an 11-item Loneliness Scale and body mass index was calculated from technician measured height and weight. Our primary outcome is high sensitivity CRP (hsCRP). Survey-weighted logistic regression models were used to test whether loneliness and BMI category are independent predictors of CRP after adjusting for demographics and other inflammatory-related covariates. In the fully adjusted model for men, obesity (OR = 2.36, p < .0001) was associated with increased odds of hsCRP >3.0. Among females, being overweight (OR = 1.75, p < .0001) or obese (OR = 4.01, p < .0001) were associated increased odds of hsCRP>3.0. Among both men and women, results from fully adjusted models indicated that loneliness was not associated with clinically elevated hsCRP (OR = 1.34, p = .0535; OR = 0.97, p = 0.6776, respectively).

## Introduction

Obesity is a leading cause of cardiovascular disease (CVD) [1]. Numerous mechanisms have been hypothesized to mediate the relationship between obesity and CVD, and inflammatory markers are now considered important pathogenic mechanisms in the initiation and progression of CVD [2]. Specifically, C-reactive protein (CRP) has emerged not only as a powerful predictor of CVD but also as a possible mechanism in the relationship between obesity and CVD [3, 4]. In addition to obesity, loneliness—the distressing feeling that accompanies the perception that one’s social needs are not being met by the quantity or quality of one’s social relationships [5–7] is a key risk factor for CVD mortality [8], comparable to smoking and other well-established risk factors for mortality [9]. And like obesity, loneliness (and similar social constructs including social isolation) is associated with elevated CRP [8].

While obesity and loneliness have been studied as separate risk factors for CRP, it is uncertain whether they operate as independent risk factors for clinically elevated CRP. Thus, the purpose of this research is to examine the independent and combined effects of loneliness and obesity on elevated CRP in older adults. We also test for interactive effects, which would indicate that the association between obesity and high sensitivity CRP **(**hsCRP) is modified among individuals who report being lonely (and vice versa). The potential additive effects of obesity and loneliness on risk of hsCRP is important as obese individuals may be especially at risk of social adversity and loneliness, predisposing them to the health consequences associated with these experiences [10].

## Methods

Data come from the RAND Health and Retirement Study (HRS) dataset and specifically from participants who completed the face-to-face interviews in the 2008 and 2010 waves of the HRS. The RAND HRS dataset is a cleaned and processed version of the raw HRS data funded by the National Institute on Aging (NIA) and the Social Security Administration [11]. The HRS is an NIA sponsored biennial longitudinal health interview survey conducted by the University of Michigan representative of non-institutionalized adults over the age of 50 age that collects information on their economic, health, marital, and family status [12] grant number NIA U01AG009740). Data included in the present analysis come from respondents who completed the enhanced face-to-face interview, which include objective measurement of height and weight and hsCRP. Only half of the 2008 and 2010 samples received the enhanced face-to-face interview. Thus, the sample of 10,912 participants (6,428 females and 4,484 males) was obtained by combining data from the 2008 and 2010 waves. Because gender differences have been reported in studies of social factors and CRP (e.g. [13] we stratified analyses by gender. Additionally, results from a likelihood ratio test indicated that a model predicting the odds of hsCRP >3.0 as a function of sex, loneliness, and weight classification, and allowing for interactions between sex and weight classification and sex and loneliness, had a significantly better fit than the model without interactions (X^2 =^ 43.11, d.f. = 3, p < .0001).

## Measures

Our primary outcome of interest is high sensitivity CRP (hsCRP), assayed from a dried blood spot using a sandwich ELISA protocol. A detailed description of the hsCRP assay used and detection limits is available online [14]. For the primary analyses, hsCRP was dichotomized into values < 3.0 ug/L and ≥ 3.0 ug/L, in line with the relative risk categories defined in the combined American Heart Association/Centers for Disease Control scientific statement, with hsCRP values greater than 3.0 indicating a greater elevated risk [15]. As a sensitivity test, we also tested whether a continuous measure of hsCRP was associated with BMI category and loneliness. For these analyses, hsCRP values were log transformed to reduce skewness (from 10.4 to 0.08).

Our two predictors of interest are loneliness and body mass index (BMI). Loneliness was measured using an 11-item scale designed for large surveys such as the HRS and based on a 3-item scale developed by [16]. Each item had three categories (e.g., How much of the time do you feel you lack companionship; 1 = often, 2 = some of the time, 3 = hardly ever or never), and responses to all items were averaged and reverse coded as needed such that higher scores indicated greater loneliness. Body mass index was calculated from technician measured height and weight and categorized as underweight (<18.5) normal weight (18.5–24.9), overweight (25.0–29.9) and obese (≥30.0). Underweight individuals were not included in the analysis (n = 143). An additional eight participants were removed from analysis due to physiologically implausible values for height (<10 inches) and one participant for weight (21lbs.).

Covariates included in analyses that may confound the relationship between obesity/loneliness and hsCRP were age (measured in years), marital status (married or partnered vs. not), race/ethnicity (non-Hispanic black, non-Hispanic white, Hispanic, and other) education (years), smoking status (current smoker vs. not), and presence of disease associated with inflammatory markers (diabetes, chronic lung disease, heart disease, and arthritis). Regarding age, preliminary results indicated that an age-squared term was not significant in models for either men or women and is not included in the final reported models.

### Analytic models

First, nationally representative descriptive statistics are computed using HRS-calculated survey weights appropriate for analyses using laboratory data from individual participants. Second, we conduct a series of logistic regression models stratified by sex to test whether loneliness and obesity are both significantly associated with clinically elevated hsCRP after adjusting for demographics and other risk factors. Model 1 estimates the likelihood of clinically elevated hsCRP with only BMI category (reference group: normal BMI) in the model; model 2 estimates the likelihood with only loneliness category (reference group: rarely lonely); model 3 includes both loneliness and BMI category; and model 4 is adjusted by demographics and health status including smoking status. To test for an interaction between loneliness and obesity, we include their interactive term in model 4.

## Results

Table 1 presents weighted descriptive statistics for the sample with complete data on hsCRP. Mean hsCRP levels among men and women were 2.60 ug/L and 3.0 ug/L, respectively. A large proportion of men and women participants were obese (41.6% and 42.6%, respectively).

**Table 1:** Descriptive statistics (weighted) of the analytic sample (years 2008–2010).

	Men		Women	
	Percent	N miss.	Percent	N miss.
Unweighted N, no.	4,484		6,428	
C-reactive protein, ug/L (CRP)		0		0
Mean CRP	2.6	-	3.0	-
<2.0	68.6	-	59.4	-
2.0–2.99	11.8	-	13.2	-
≥ 3.0	19.5	-	27.4	-
Mean loneliness (1–3)	1.5	-	1.5	-
Loneliness		570		770
1.0 (Hardly ever lonely)	12.8	-	16.3	-
1.0–1.99 (Sometimes lonely)	68.1	-	67.0	-
≥ 2.0 (Often lonely)	19.1	-	16.8	-
Body mass index		0		0
Mean BMI (underweight excluded)	29.7		29.7	
18.5-0-24.9 (normal)	16.8	-	26.0	-
25.0–29.9(over)	41.6	-	31.3	-
≥ 30.0 (obese)	41.6	-	42.6	-
Age, years	66.7	0	67.6	0
Race		2		1
Non-Hispanic white	82.7	-	80.3	-
Non-Hispanic black	7.8	-	9.4	-
Hispanic	6.9	-	7.7	-
Other	2.7	-	2.5	-
Education, years.	13.3	8	12.8	10
Married/Partner	78.0	0	56.7	1
Current Smoker	14.2	29	12.4	22
Disease				
Diabetes	21.1	3	18.7	7
Chronic lung disease	8.7	7	10.7	7
Heart disease	27.0	3	20.0	12
Arthritis	51.4	6	65.6	4

Approximately 19.1% of men and 16.8% of women had a mean loneliness score ≥ 2.0, indicating a loneliness score roughly equivalent to “often” being lonely. Among women who were obese, approximately 18% reported often being lonely, compared to 16% of women categorized as normal weight.

**Fig 1:**
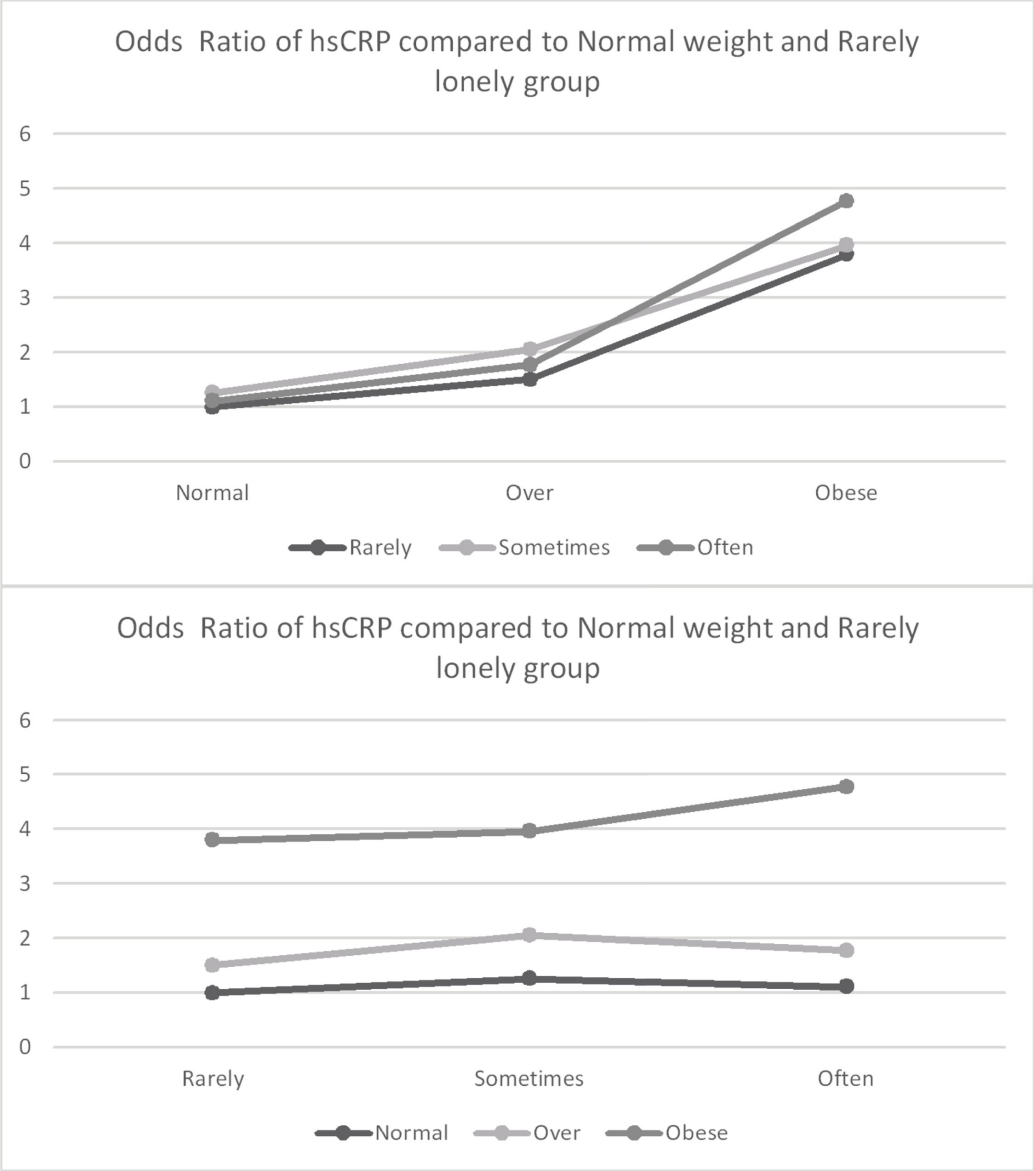
Odds ratio of hsCRP compared to normal weight and rarely lonely group–Men and women combined. Estimation was adjusted by Age, Female, Black, Education, Marital Status, Smoking status, and Disease (Diabetes, Chronic lung disease, Heart disease, Arthritis).

**Fig 2:**
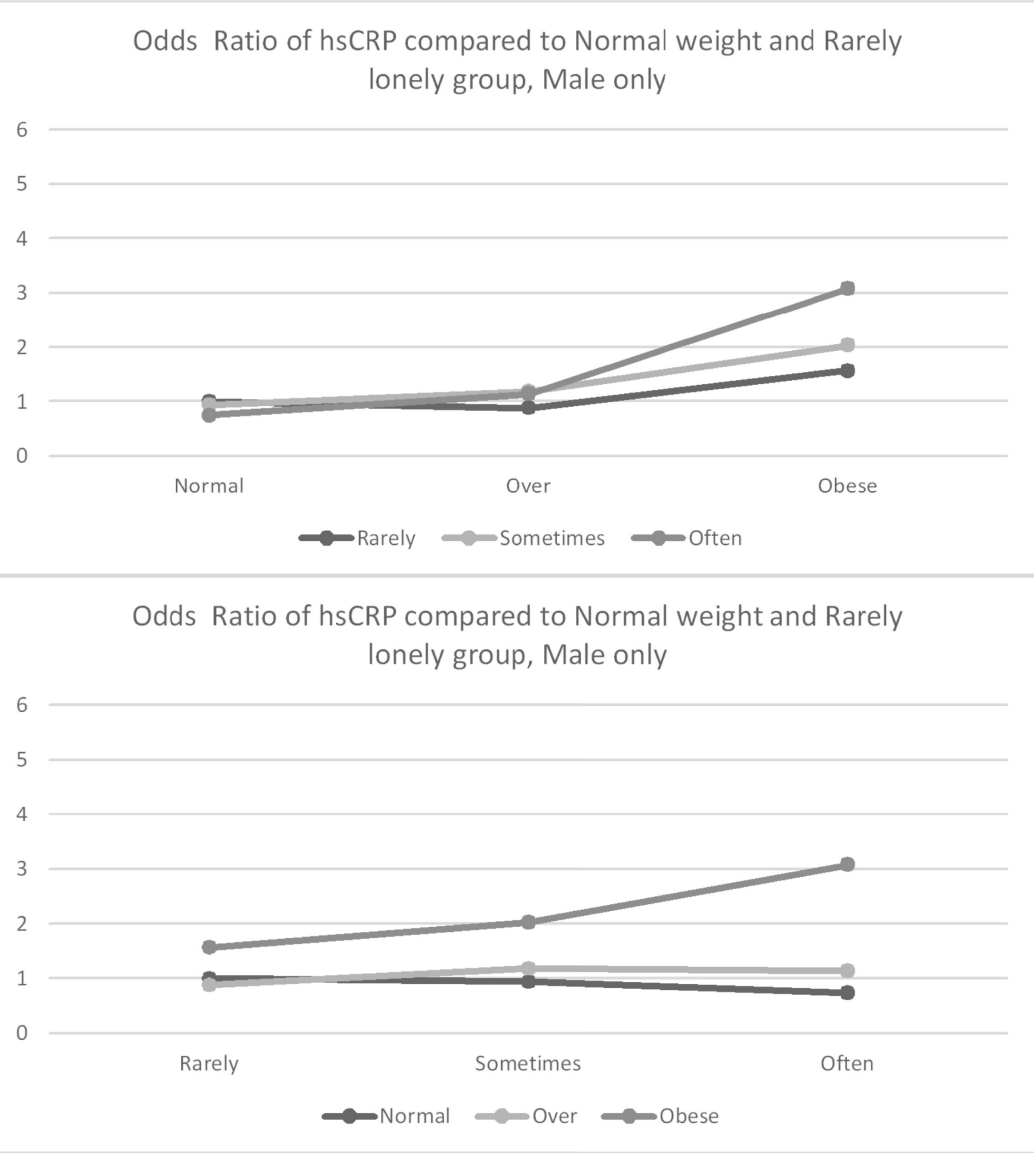
Odds ratio of hsCRP compared to normal weight and rarely lonely group–men only. Estimation was adjusted by Age, Black, Education, Marital Status, Smoking status, and Disease (Diabetes, Chronic lung disease, Heart disease, Arthritis).

**Fig 3:**
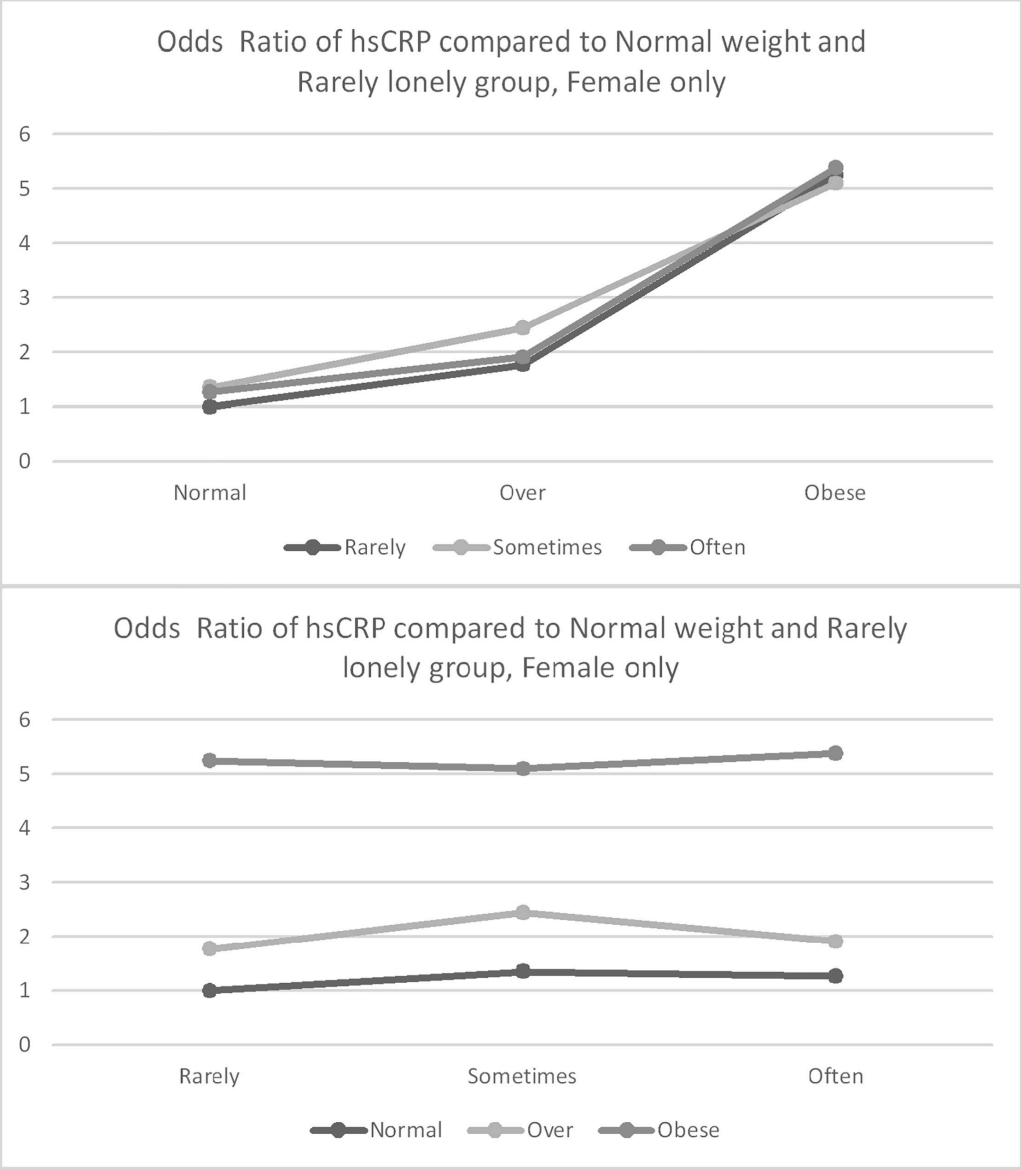
Odds ratio of hsCRP compared to normal weight and rarely lonely group–women only. Estimation was adjusted by Age, Black, Education, Marital Status, Smoking status, and Disease (Diabetes, Chronic lung disease, Heart disease, Arthritis).

Table 2 presents results from a logistic regression model predicting the likelihood of hsCRP greater than 3.0 ug/L. Fig 1 (all participants), Fig 2 (Men only) and Fig 3 (Women only) presents the associations between loneliness, obesity, and hsCRP. In the fully adjusted model for men (Model 4), obesity (OR = 2.36, p < .0001) was associated with increased odds of elevated hsCRP; however, loneliness was not associated with increased odds of elevated hsCRP (OR = 1.34, p = .0536). Among females, being overweight (OR = 1.75, p < .0001) and obese (OR = 4.01, p < .0001) were associated with increased odds of elevated hsCRP. Among women, loneliness was not associated with elevated hsCRP (OR = 0.97, p = 0.6776), and there was no evidence of an interaction between obesity and loneliness. However, the association between obesity and clinically elevated hsCRP was stronger among men who reported higher loneliness than among men who reported being less frequently lonely (b = 0.70, p = 0.0477).

**Table 2:** Adjusted odds ratios (95% confidence intervals) for clinically raised C-reactive protein (CRP) concentrations (years 2008–2010).

ALL	Model 1	p	Model 2	p	Model 3	p	Model 4**	p
Body mass index*								
Overweight	1.46(1.22–1.74)	< .0001	-		1.45(1.22–1.74)	< .0001	1.62(1.35–1.93)	< .0001
Obese	3.22(2.68–3.89)	< .0001	-		3.21(2.67–3.87)	< .0001	3.41(2.79–4.16)	< .0001
Loneliness*								
Sometimes	-		1.24(1.05–1.46)	0.0144	1.22(1.03–1.45)	0.0238	1.17(0.98–1.41)	0.0871
Often	-		1.44(1.16–1.79)	0.0014	1.38(1.11–1.73)	0.0049	1.14(0.97–1.56)	0.0906
Men	Model 1	p	Model 2	p	Model 3	p	Model 4**	p
Body mass index*								
Overweight	1.10(0.83–1.45)	0.4935	-		1.10(0.83–1.46)	0.5091	1.26(0.96–1.66)	0.0999
Obese	1.99(1.45–2.73)	< .0001	-		1.97(1.43–2.72)	< .0001	2.37(1.73–3.25)	< .0001
Loneliness*								
Sometimes	-		1.36(1.02–1.81)	0.0414	1.35(1.01–1.81)	0.0493	1.23(0.91–1.65)	0.1705
Often	-		1.72(1.17–2.53)	0.0063	1.68(1.14–2.48)	0.0097	1.44(0.96–2.14)	0.0747
Women	Model 1	p	Model 2	p	Model 3		Model 4**	p
Body mass index*								
Overweight	1.82(1.45–2.29)	< .0001	-		1.82(1.45–2.30)	< .0001	1.76(1.40–2.22)	< .0001
Obese	4.48(3.71–5.40)	< .0001	-		4.46(3.70–5.38)	< .0001	4.03(3.25–4.99)	< .0001
Loneliness*								
Sometimes	-		1.24(1.01–1.52)	0.0448	1.22(0.99–1.51)	0.0647	1.16(0.93–1.44)	0.1897
Often	-		1.38(1.11–1.72)	0.0053	1.32(1.05–1.66)	0.0180	1.11(0.87–1.42)	0.3840

*Reference group for BMI is normal weight. Reference group for Loneliness is ‘Rarely lonely’.

**Model 4 is adjusted by Age, Female, Black, Education, Marital Status, Smoking status, and Disease (Diabetes, Chronic lung disease, Heart disease, Arthritis). For the combined model (men + women), statistically significant adjustors were: female (p < 0.001), white (p <0.001), education (p = 0.017), smoking status (p <0.001), diabetes (p = 0.004), lung (p<0.001), and heart diseases (p = 0.048). In the male only model, age (p<0.001), white (p = 0.007), smoking status (p<0.001), and lung disease (p = 0.004) were statistically significant adjustors. For the female only model, white (p<0.001), smoking status (p<0.019), diabetes (p = 0.003), and lung diseases (p<0.001) were statistically significant adjustors.

In the sensitivity analysis predicting log-transformed hsCRP (full results not shown), loneliness was significantly associated with increased hsCRP among men (b = 0.16, p = .0168). Among men, being overweight (b = 0.34, p < .0001) or obese (b = 0.76, p < .0001) were also associated with increased hsCRP. Because hsCRP is log-transformed, regression coefficients can be interpreted as the estimated percent change in log hsCRP per one-unit change in the independent variable. Thus, an individual with normal BMI and who report being “sometimes lonely” has a 16% increase in hsCRP relative to the same individual who report being “rarely lonely”. An individual classified as overweight and who is “sometimes lonely” is estimated to have hsCRP levels 50% higher than an individual classified as normal weight and who report being rarely lonely.

## Discussion

To our knowledge, this is the first study to consider obesity and loneliness as additive risk factors for elevated C-reactive protein. Obesity and loneliness have both been identified as predictors of elevated CRP, which in turn, is a risk factor for CVD and coronary heart disease. Our findings are consistent with previous research showing a relationship between weight classification and hsCRP [17]. For both men and women, being obese was associated with clinically elevated hsCRP. Given evidence that individuals who are obese may be at increased risk of loneliness, we sought to test whether obesity and loneliness operated to additively increase the risk of clinically elevated hsCRP [18]. Overall, we found little evidence that it was the case, as loneliness did not confer additional risks of elevated hsCRP. These findings are in contrast with studies on social isolation (i.e., objective and quantifiable reflection of reduced social network size and paucity of social contact [19]) indicating that having few close relationships or social ties is an independent risk factor of both high levels of CRP and coronary heart disease death (e.g., [8, 19]). Researchers have long debated whether the health effects of social isolation result from the objective lack of social contacts or from the subjective experience of being lonely [9, 19–24]. Our analyses suggest that the relationship between obesity and hsCRP is not explained by subjective loneliness as a pathway through which obesity may lead to clinically elevated hsCRP, although we noted that when hsCRP was treated as a continuous variable in sensitivity analyses, loneliness was significantly associated with hsCRP in men.

Given the cross-sectional nature of the data, we cannot make definitive conclusions regarding the directionality of the associations; the finding that obese men who reported feeling lonely more frequently had elevated hsCRP levels could be explained by reverse causality, whereby men who are more lonely are more likely to gain weight [25] or men with lower hsCRP levels feel less lonely (see [26] for a similar example of reverse causality).

It is also important to acknowledge that levels of the inflammatory marker CRP assessed from a single venipuncture do not fully capture the dynamic processes involved in inflammation, or make it possible to distinguish acute from chronic inflammation. This is important because the adverse effects of loneliness operate slowly over time through multiple mechanistic pathways in both the development and progression of disease.

Prospective studies are needed to fully delineate whether and how social relationships contribute to mechanisms linking inflammatory markers to cardiovascular health, morbidity and mortality. Studying direct and indirect pathways between a wide and specific spectrum of social factors, health and all-cause mortality using a life course approach is essential because social relations have been shown to influence multiple mechanistic pathways in both the development and progression of disease [27, 28]. These questions are important to identify the most effective strategies to buffer the effects of adversity and risk factors on health. To the extent that these strategies are successful in influencing biomarkers, this work would provide evidence that social processes are acting on health risks in a causal fashion.

## References

[1] Poirier P, Giles TD, Bray GA, Hong Y, Stern JS, Pi-Sunyer FX, et al Obesity and cardiovascular disease: pathophysiology, evaluation, and effect of weight loss. Arterioscler Thromb Vasc Biol. 2006;26(5):968–76. 10.1161/01.ATV.0000216787.85457.f3 . 16627822 10.1161/01.ATV.0000216787.85457.f3

[2] Libby P, Ridker PM, Hansson GK, Leducq Transatlantic Network on A. Inflammation in atherosclerosis: from pathophysiology to practice. J Am Coll Cardiol. 2009;54(23):2129–38. 10.1016/j.jacc.2009.09.009 ; PubMed Central PMCID: PMCPMC2834169. 19942084 10.1016/j.jacc.2009.09.009 PMC2834169

[3] Grad E, Danenberg HD. C-reactive protein and atherothrombosis: Cause or effect? Blood Rev. 2013;27(1):23–9. 10.1016/j.blre.2012.12.001 . 23266251 10.1016/j.blre.2012.12.001

[4] Choi J, Joseph L, Pilote L. Obesity and C-reactive protein in various populations: a systematic review and meta-analysis. Obes Rev. 2013;14(3):232–44. 10.1111/obr.12003 . 23171381 10.1111/obr.12003

[5] Hawkley LC, Hughes ME, Waite LJ, Masi CM, Thisted RA, Cacioppo JT. From social structural factors to perceptions of relationship quality and loneliness: the Chicago health, aging, and social relations study. J Gerontol B Psychol Sci Soc Sci. 2008;63(6):S375–84. ; PubMed Central PMCID: PMCPMC2769562. 19092047 10.1093/geronb/63.6.s375 PMC2769562

[6] Weeks DJ. A review of loneliness concepts, with particular reference to old age. International Journal of Geriatric Psychiatry. 1994;9:345–55.

[7] Wheeler L, Reis H, Nezlek J. Loneliness, social interaction, and sex roles. J Pers Soc Psychol. 1983;45(4):943–53. . 6631669 10.1037//0022-3514.45.4.943

[8] Heffner KL, Waring ME, Roberts MB, Eaton CB, Gramling R. Social isolation, C-reactive protein, and coronary heart disease mortality among community-dwelling adults. Soc Sci Med. 2011;72(9):1482–8. Epub 2011/04/16. 10.1016/j.socscimed.2011.03.016 ; PubMed Central PMCID: PMCPMC3090468. 21492978 10.1016/j.socscimed.2011.03.016 PMC3090468

[9] Cacioppo JT, Cacioppo S. Social Relationships and Health: The Toxic Effects of Perceived Social Isolation. Soc Personal Psychol Compass. 2014;8(2):58–72. 10.1111/spc3.12087 ; PubMed Central PMCID: PMCPMC4021390. 24839458 10.1111/spc3.12087 PMC4021390

[10] Latner JD, O' Brien KS, Durso LE, Brinkman LA, MacDonald T. Weighing obesity stigma: the relative strength of different forms of bias. International Journal of Obesity. 2008;32:1145–52. 10.1038/ijo.2008.53 18414421 10.1038/ijo.2008.53

[11] RAND. RAND HRS Data, Version M. Santa Monica, CA.: RAND Center for the Study of Aging, with funding from the National Institute on Aging and the Social Security Administration.; 2013.

[12] National Institute on Aging. Growing Older in America—The Health and Retirement Study. 2007.

[13] Ford ES, Loucks EB, Berkman LF. Social integration and concentrations of C-reactive protein among US adults. Ann Epidemiol. 2006;16(2):78–84. 10.1016/j.annepidem.2005.08.005 . 16271297 10.1016/j.annepidem.2005.08.005

[14] Crimmins EM, Guyer H, Langa KM, Ofstedal MB, Wallace RB, Weir DR. Documentation of Physical Measures, Anthropometrics and Blood Pressure in the Health and Retirement Study. Retrieved from http://hrsonline.isr.umich.edu/sitedocs/userg/dr-011.pdf: 2008.

[15] Pearson TA, Mensah GA, Alexander RW, Anderson JL, Cannon RO 3rd, Criqui M, et al Markers of inflammation and cardiovascular disease: application to clinical and public health practice: A statement for healthcare professionals from the Centers for Disease Control and Prevention and the American Heart Association. Circulation. 2003;107(3):499–511. . 12551878 10.1161/01.cir.0000052939.59093.45

[16] Hughes ME, Waite LJ, Hawkley LC, Cacioppo JT. A short scale for measuring loneliness in large surveys: results from two population-based studies. Research on Aging. 2004:655–72. 10.1177/0164027504268574 18504506 10.1177/0164027504268574 PMC2394670

[17] Visser M, Bouter LM, McQuillan GM, Wener MH, Harris TB. Elevated C-reactive protein levels in overweight and obese adults. JAMA. 1999;282(22):2131–5. . 10591334 10.1001/jama.282.22.2131

[18] Schumaker JF, Krejci RC, Small L, Sargent RG. Experience of loneliness by obese individuals. Psychol Rep. 1985;57(3 Pt 2):1147–54. 10.2466/pr0.1985.57.3f.1147 . 4095228 10.2466/pr0.1985.57.3f.1147

[19] Steptoe A, Shankar A, Demakakos P, Wardle J. Social isolation, loneliness, and all-cause mortality in older men and women. Proc Natl Acad Sci U S A. 2013;110(15):5797–801. 10.1073/pnas.1219686110 ; PubMed Central PMCID: PMCPMC3625264. 23530191 10.1073/pnas.1219686110 PMC3625264

[20] Cacioppo JT, Hawkley LC. Social isolation and health, with an emphasis on underlying mechanisms. Perspect Biol Med. 2003;46(3 Suppl):S39–52. . 14563073

[21] Cole SW, Hawkley LC, Arevalo JM, Sung CY, Rose RM, Cacioppo JT. Social regulation of gene expression in human leukocytes. Genome Biol. 2007;8(9):R189 10.1186/gb-2007-8-9-r189 ; PubMed Central PMCID: PMCPMC2375027. 17854483 10.1186/gb-2007-8-9-r189 PMC2375027

[22] Holt-Lunstad J, Smith TB, Layton JB. Social relationships and mortality risk: A meta-analytic review. PLoS Medicine. 2010;7(7):e1000316 10.1371/journal.pmed.1000316 20668659 10.1371/journal.pmed.1000316 PMC2910600

[23] Loucks EB, Berkman LF, Gruenewald TL, Seeman TE. Relation of social integration to inflammatory marker concentrations in men and women 70 to 79 years. Am J Cardiol. 2006;97(7):1010–6. 10.1016/j.amjcard.2005.10.043 . 16563907 10.1016/j.amjcard.2005.10.043

[24] Loucks EB, Sullivan LM, D'Agostino RB Sr., Larson MG, Berkman LF, Benjamin EJ. Social networks and inflammatory markers in the Framingham Heart Study. J Biosoc Sci. 2006;38(6):835–42. 10.1017/S0021932005001203 . 16441967 10.1017/S0021932005001203

[25] Kershaw KN, Hankinson AL, Liu K, Reis JP, Lewis CE, Loria CM, et al Social relationships and longitudinal changes in body mass index and waist circumference: the coronary artery risk development in young adults study. Am J Epidemiol. 2014;179(5):567–75. 10.1093/aje/kwt311 ; PubMed Central PMCID: PMCPMC3927980. 24389018 10.1093/aje/kwt311 PMC3927980

[26] Das A. Psychosocial distress and inflammation: Which way does causality flow? Soc Sci Med. 2016;170:1–8. 10.1016/j.socscimed.2016.10.001 . 27728857 10.1016/j.socscimed.2016.10.001

[27] Holt-Lunstad J, Smith TB. Loneliness and social isolation as risk factors for CVD: implications for evidence-based patient care and scientific inquiry. Heart. 2016;102(13):987–9. 10.1136/heartjnl-2015-309242 ; PubMed Central PMCID: PMCPMC4941164. 27091845 10.1136/heartjnl-2015-309242 PMC4941164

[28] Holt-Lunstad J, Smith TB, Baker M, Harris T, Stephenson D. Loneliness and social isolation as risk factors for mortality: a meta-analytic review. Perspect Psychol Sci. 2015;10(2):227–37. 10.1177/1745691614568352 . 25910392 10.1177/1745691614568352

